# A Serious Game (Health Unit in Focus) for Enhancing Undergraduate Education on Older Adults’ Health: Design and Validation Study

**DOI:** 10.2196/66289

**Published:** 2025-11-04

**Authors:** Yuri Gustavo de Sousa Barbalho, Calliandra Maria de Souza Silva, Carla Sílvia Neves da Nora Fernandes, Raíza Rana de Souza Lima Trombini, Patrícia Littig Melo, Aline Farias de Oliveira, Alayne Larissa Martins Pereira, Alessandro de Oliveira Silva, Luciano Ramos de Lima, Marina Morato Stival, Diana Lúcia Moura Pinho, Silvana Schwerz Funghetto

**Affiliations:** 1 Faculty of Health Sciences and Technologies, University of Brasília, University Campus - Metropolitan Center, Ceilândia Sul, Brasília, Brazil, 55 61991127116; 2 School of Health, Polytechnic Institute of Viana do Castelo, Viana do Castelo, Portugal; 3 Aditgames – Association for Innovation, Technologies and Games in Health, Porto, Portugal; 4 Physical Education and Medicine Department, University Center of Brasilia, Brasília, Brazil; 5 Nursing Department, University of Brasília, Brasilia, Brazil

**Keywords:** serious games, nursing education, aged, older adults, design, validation

## Abstract

**Background:**

Population aging underlines the critical need to improve health professional training to adequately care for adults aged >60 years. Developing educational resources to support academics and professionals presents a valuable opportunity to enhance understanding of health conditions and improve clinical management. Serious games are designed to develop teaching, training, and learning skills. Their use in the educational setting is warranted, as they integrate digital aspects and gamification to create a playful experience for content acquisition. Deepening this theme in nursing education will improve assistance to the older adult population, leading to more qualified care based on gerontological practices and comprehensive health care for older adults.

**Objective:**

This study aims to develop and validate a serious game on older adult health for undergraduate nursing students.

**Methods:**

This quantitative and descriptive methodological study, conducted between February 2023 and December 2023 at a public university in the Federal District of Brazil, involved the active participation of 27 undergraduate nursing students in their eighth to tenth semesters. The game, Health Unit in Focus (HUF), was developed and validated with their input. It features 75 clinical cases distributed across 3 themes: pharmacology, metabolic syndrome, and semiology. Of the 40 students initially enrolled, 27 completed the study. The app was validated using the System Usability Scale and student feedback, and the results were reported following the Game-Based Intervention Reporting Guidelines (GAMING).

**Results:**

The participants had a mean age of 22.67 (SD 1.44) years, were mostly female (20/27, 74%), and were in their eighth semester (26/27, 96%). The game received an average System Usability Scale score of 85.75 (median 86.57), classified as excellent, as all evaluated items scored >75. Participants considered the game easy to use; accessible; practical; and rich in well-founded, useful content. This high usability score, coupled with the overwhelmingly positive feedback from the students, instills confidence in the game’s effectiveness. Furthermore, 100% (27/27) of students agreed that learning through games is effective and expressed interest in incorporating more interactive games into their training. The serious game HUF showed good usability, as its overall score was “excellent,” with its highest score in the odd-numbered items that addressed the positive aspects identified in the analysis.

**Conclusions:**

The serious game HUF is not just a valid and reliable tool for training nursing students but also an engaging and interactive approach to learning. Its ability to captivate and involve students in the learning process is a testament to its potential to revolutionize nursing education. It is essential that the development of new methodological resources, such as serious games, be based on scientific evidence to guarantee greater reliability and success in achieving their established objectives.

## Introduction

### Background

On the global stage, the health concerns of older adults (aged >60 y) have gained importance driven by population aging and the epidemiological transition. This transition reflects a shift in the pattern of mortality and disease, with a growing prevalence of chronic noncommunicable diseases in older age groups [1]. United Nations data show a 3-fold increase in older adults between 1980 and 2022. By 2023, this demographic was expected to reach 994 million, accounting for 12% of the global population. Over the next 3 decades, the older adult population worldwide is projected to more than double, reaching >1.5 billion people by midcentury [2].

Under these circumstances, health practices should prioritize promoting active aging by enhancing quality of life, supporting health maintenance, and preventing disease [3]. In the 2000s, the World Health Organization introduced “active aging” policies that addressed various determinants, including health, social, and economic factors, while also emphasizing the need for adequately trained professionals to support and assist this population [4].

In this sense, developing educational resources to support academics and professionals presents a valuable opportunity to enhance understanding of health conditions and improve clinical management. These resources can take various forms, such as guides, brochures, applications, and games, providing information that can effectively optimize the teaching-learning process in health [5].

Serious games are designed to develop teaching, training, and learning skills, with the first records dating back to the 1970s [6]. These days, they are most typically used in digital games, although they can also be used in physical games. Their use in the educational setting is warranted because they integrate digital aspects and gamification to create a playful content acquisition experience [7]. In recent years, they have been widely applied in health education [8,9].

One study compared the effects of traditional lectures with a serious game on nursing students’ knowledge and skills in assessing patients with burns, finding a significant improvement in educational outcomes among those who used the game [10]. Similarly, another study explored the effects of using a serious game in a home environment, reporting an improvement in theoretical knowledge related to the game’s central theme [11].

### Objectives

This study introduces a novel approach to teaching issues related to older adult health conditions commonly encountered in primary health care (PHC). The serious game, designed for any mobile device, offers content tailored to the target population and is adaptable to students’ daily lives. At the time of this research, our game was the only application identified that integrated these specific characteristics, making it a unique and potentially impactful tool in nursing education.

Deepening this theme in nursing education will improve assistance to the older adult population, leading to more qualified care based on gerontological practices and comprehensive health care for older adults.

Once developed and validated, a serious learning game focused on the health of older adults with chronic noncommunicable diseases could be a strategic tool for promoting independent learning among students. Building on this potential for self-directed learning, this study, rooted in methodological development research, aims to create and validate a serious game on older adults’ health for undergraduate nursing students.

## Methods

### Study Development and Population

**Figure 1.**
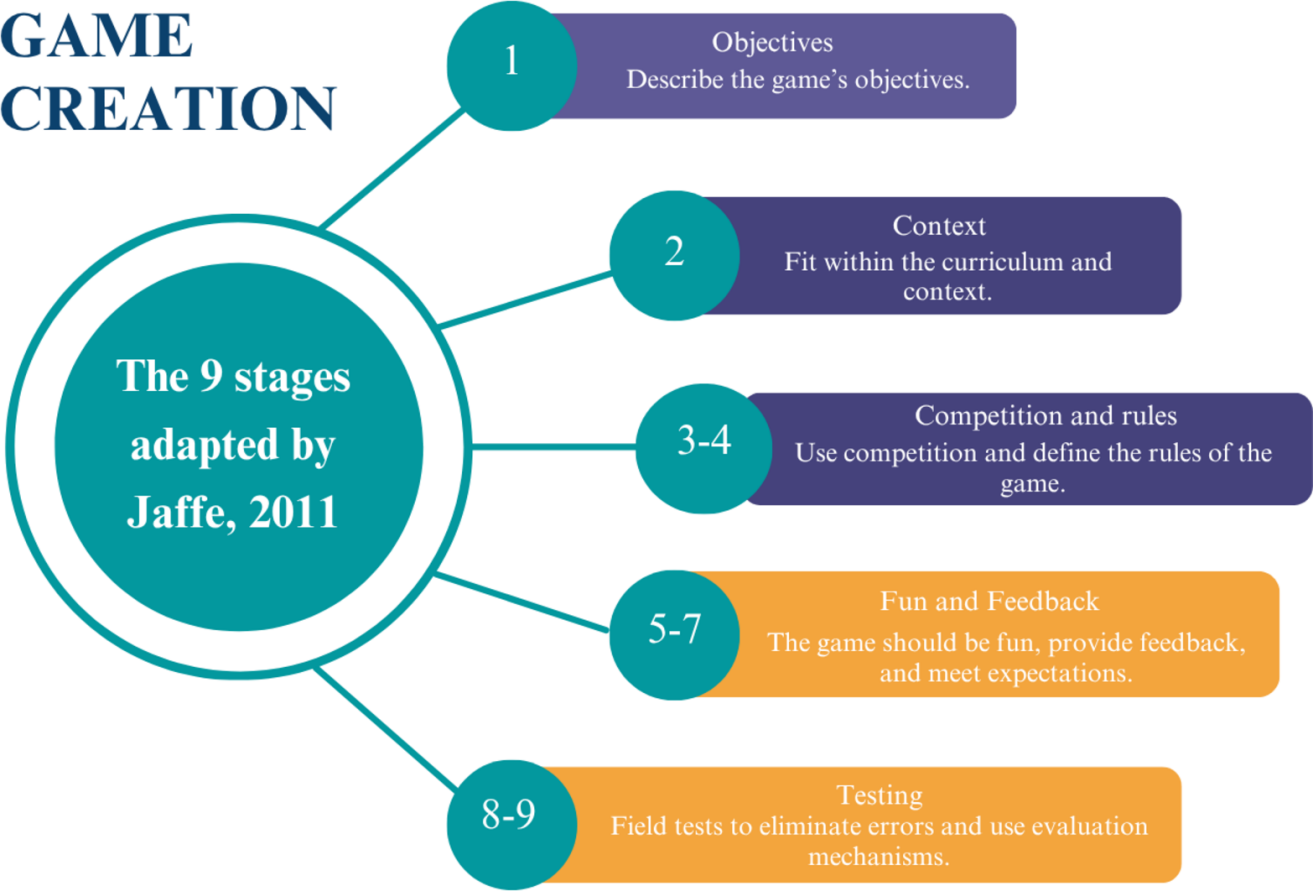
Stages of game construction (adapted from Jaffe [13]).

This methodological development study, using a quantitative and descriptive approach, was conducted at a public university in the Federal District of Brazil between February 2023 and December 2023 and was reported according to the recommendations of the Game-Based Intervention Reporting Guidelines (GAMING) [12]. The stages proposed by Jaffe [13] were used for the construction of the serious game. Figure 1 [13] describes the stages of the game’s development, testing, and validation.

In stage 1, the game’s objectives, educational aims, content, and problematizations were established. For stage 2, the context in which students would use the theme was surveyed. In stage 3, the competitive aspects were adjusted to the application’s proposal, as it is a single-player game. For the rules, in stage 4, a “help” tab was set up with all the rules and guidelines. Stage 5 aimed to integrate the gamification aspects. Stage 6, immediate feedback, was included immediately after the participants’ responses.

In stage 7, the researchers of this study analyzed and carefully studied such variables as students’ desire and expectations regarding educational games, especially because they were inserted within the academic context of the population. For evaluation, in step 8, the study conducted a pilot test with 13 undergraduate students who met the minimum knowledge requirements for game use. All participants were enrolled in a discipline that dealt with aging in health. In the last step, the support team adjusted the notes based on the evaluation from the previous step.

The themes included in the serious game were developed based on the needs of nursing undergraduates, focusing on areas of older adults’ health where students faced the greatest difficulty or expressed the most interest in deepening their knowledge. This step of assessing students’ needs had already been conducted previously. The game incorporates 75 clinical cases, with 25 cases for each of the following themes: pharmacology, metabolic syndrome, and semiology in the context of older adults’ health (Multimedia Appendix 1). All clinical cases had been previously validated by expert judges.

The cases were built and validated by experts on older adult health topics. Each case includes contextual information and a true-or-false statement, based on the most prevalent themes in the clinical context of each older adult health topic.

### Sample

After the serious game application was fully developed, its usability was evaluated by eighth-semester undergraduate nursing students at a public university. The initial sample consisted of 40 enrolled students, of which n=13 (32.5%) were excluded (10/40, 25% for not attending, 2/40, 5% for withdrawing from the course during collection, and 1/40, 2.5% for dropping out during the validation process), resulting in n=27 final participants. It is assumed that at a 95% CI, at least 70% of the experts will classify the item as appropriate. The calculation would be n=1.96^2^×0.85×0.15/0.15^2^=22 students [14]. To detect effect sizes of 0.85 with an α level of .05 and a power of 95%, a sample size of 22 participants was required. Considering the likelihood of participant dropout or ineligibility, we increased our target sample size to 27 participants. Although sample size calculations ensured adequate statistical power, future multicenter studies are recommended to strengthen external validity and account for institutional and regional educational variations.

### Data Collection Tools

Before validation, students completed a characterization questionnaire collecting information on age, sex, semester, education, year of entry into their current degree, participation in scientific initiation, prior experience with educational technologies (such as games), beliefs about the potential to learn through games, and frequency of mobile game use. The scale used for this validation was the System Usability Scale (SUS) [15]. The SUS and Educational Games Evaluation Scale were selected for their demonstrated reliability, ease of interpretation, and extensive validation in educational technology research.

This scale assesses the user’s degree of agreement using a Likert scale, ranging from “totally agree” to “totally disagree.” The final score is calculated by summing the individual contributions of each item. For even-numbered items, subtract the user’s response from 5 (5−user’s response); for odd-numbered items, subtract 1 from the user’s response (user’s response−1). The resulting values are then added and multiplied by 2.5, yielding a “user satisfaction index” ranging from 0 to 100. The score is classified as follows: worst imaginable (0‐20.5), poor (21‐38.5), average (39‐52.5), good (53‐73.5), excellent (74‐85.5), and best imaginable (86-100). The system can be further classified based on the following categories: ease of memorization (item 2), ease of comprehension (items 3, 4, 7, and 10), system efficiency (items 5, 6, and 8), user satisfaction (items 1, 4, and 9), and identification of inconsistencies (item 6) [16,17]. This instrument was printed and delivered to participants.

The serious game was evaluated using the Educational Games Evaluation Scale proposed by Savi et al [18] to assess game quality. The scale comprises 3 main components: motivation (10 questions), user experience (16 questions), and learning (3 questions), totaling 29 items. Responses are recorded on a 5-point Likert-type scale.

### Statistical Analysis

Data from the answers database were processed and analyzed using SPSS (version 20.0; IBM Corp). Statistical tests were conducted as necessary, with *P* values <.05 considered statistically significant. The Shapiro-Wilk test was used to assess normality, and the data were found to be normally distributed. Content validity was evaluated based on the questions’ relevance, purpose, and clarity of the information. Reliability was calculated by the Cronbach α index, which estimates the internal consistency of the questionnaire items [19].

The Likert-type scales have the following configurations: judgments with responses 1 and 2 characterize total disagreement and disagreement with the statement, respectively. Judgment with response 3 in the answers characterizes indecision; the student neither agrees nor disagrees with the statement. Meanwhile, judgments with responses 4 and 5 characterize agreement with and total agreement with the statement, respectively.

### Ethical Considerations

This study was rigorously designed and implemented according to the ethical guidelines established by the Declaration of Helsinki [20], with ethics approval granted by the Research Ethics Committee of the Faculty of Ceilândia at the University of Brasília (protocol 5.237.938). Participants engaged actively by downloading their match data and submitting it securely through the university’s educational e-platform. This platform provided a protected digital storage area exclusively accessible to the research team, ensuring participant confidentiality.

Prior to enrollment, all potential participants received clear and detailed explanations of the study’s purpose, methods, and procedures, as well as the specific types of data to be collected. Written informed consent was voluntarily obtained from each participant, emphasizing their autonomy and freedom to withdraw from the research at any point without any repercussions or adverse outcomes.

To protect the participants’ right to privacy, personal identifiers were safeguarded in secure, password-protected digital files. During data analysis and the reporting of results, only anonymized or pseudonymized information was used. Specifically, each nursing student participant was assigned a unique numeric identifier according to the sequential order of their questionnaire submissions, ensuring complete anonymity throughout the research process.

Participants received no financial incentives for their involvement, aligning with ethical standards promoting voluntary and unbiased participation. Additionally, considerable care was taken to prevent any potential identification through the publication or supplementary materials associated with this study. In instances where the use of potentially identifiable images was unavoidable, explicit written consent was obtained from the concerned individuals. However, no identifiable images were ultimately included in this paper or its supplementary documentation, thereby reinforcing the commitment to participants’ confidentiality and ethical research conduct.

## Results

### Serious Game: Health Unit in Focus

The final product of this study is a serious game app called Health Unit in Focus (HUF) for mobile devices. HUF is a single-player question-and-answer game composed of clinical cases with online and offline functionality. On its home screen are fields for log-in and password if the user is already registered and a field for creating a new account.

Also, on its home screen, the player can access the game’s established areas: pharmacy for pharmacology cases, laboratory for metabolic syndrome, and consulting room for semiology. The player can also consult solved cases and other players’ achievements in the archive and secretary areas. Furthermore, a help tab provides information on game areas, how to play, emergencies, content review, achievements, and profiles.

**Figure 2.**
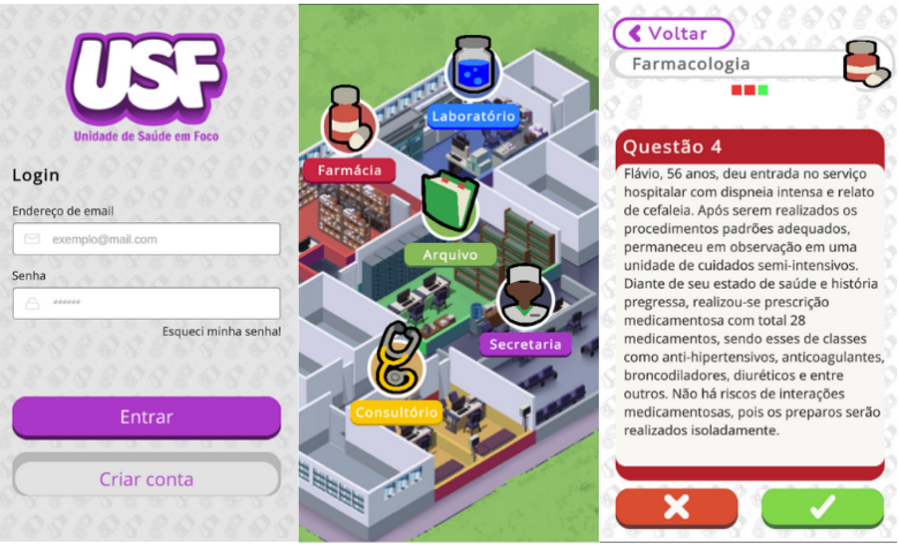
Tabs of the Health Unit in Focus app.

Upon entering the game area, participants must respond to a clinical case on their chosen topic. These cases are categorized as easy, medium, or difficult, and participants must complete the initial levels to advance to more challenging ones. Immediate feedback is provided after each response, explaining the correct answer. Emergency scenarios will also appear during gameplay, requiring the participant to respond within 60 seconds. Figure 2 presents some of the tabs from the HUF application.

### Stages in the Construction of the Serious Game

The game’s scenarios focus on older adults’ health issues within the PHC context, combining technological resources with gamification and innovation to create an engaging and effective learning experience [21]. The construction of the serious game involved 9 stages.

#### Stage 1: Developing and Describing the Game’s Specific Objectives

According to Jaffe [13], developing an educational game requires determining the content; problematizing it; and setting clear, context-aligned objectives. Accordingly, the application aimed to enhance knowledge of older adults’ health in PHC clinical contexts by engaging students with problem-based content on pharmacology, chronic noncommunicable diseases (metabolic syndrome), and semiology.

The game development explicitly integrated pedagogical theories, such as experiential learning and self-directed learning, to ensure that clinical cases and interactive elements facilitated deeper cognitive engagement and autonomous knowledge construction.

#### Stage 2: The Context Is Inserted (Contextual Integration)

This serious game model was intended for use by undergraduate nursing students during their final year of study.

#### Stage 3: Competitive Space

As a single-player game, HUF included a feature that allows users to compare their achievements, introducing an element of competition.

#### Stage 4: Defining the Game Rules

The game included detailed rule descriptions, gameplay mechanics, game areas, content review, and how to earn new achievements. Immediate feedback and emergency scenarios were purposefully designed to replicate the urgency of clinical decision-making and foster real-time critical thinking skills directly related to clinical practice.

#### Stage 5: Fun

Gamification aspects commonly seen in games and applications were incorporated to enhance engagement [22].

#### Stage 6: Immediate Feedback

Upon evaluating a clinical case and selecting a response, the user receives immediate feedback on the success or failure of their choice.

#### Stage 7: Meeting the Needs of the Participants

The themes in HUF are designed to address the target audience’s needs, considering factors such as age, gender, culture, and academic level.

#### Stages 8 and 9: Testing and Evaluation Mechanisms

This research involves testing the application prototype and assessing its usability with undergraduate nursing students.

During stages 8 and 9, a total of 13 undergraduate nursing students (mean age 22 years; 9/13, 69% female) participated in the study. Their profile closely matched the final target audience. The students assessed the game’s quality using a scale developed by Savi et al [18], which was divided into 3 categories: motivation, user experience, and learning. The results indicate that most statements received a satisfactory average in student evaluations, suggesting that the game can be effectively used as an educational tool based on the evaluated points (Multimedia Appendix 2).

Four items positively highlighted the motivation and user experience categories, with average ratings of 4.6 and 4.8, respectively. These items pertained to the game’s relevance, recommendation to friends, interest in playing again, and ease of learning. The learning domain also stood out, with high averages in 2 of its 3 items. These statements focused on the game’s contribution to knowledge and its efficiency in learning.

### Usability Validation (Sample)

The sample that validated the game’s usability closely matches the desired profile of the serious game’s end users. It comprised 27 undergraduate nursing students, primarily women, all in the eighth semester of their undergraduate degree.

The need for digital literacy was not a concern for these researchers, as the students already had previous experience of using digital platforms in previous subjects, such as Mentimeter and Kahoot, among others. This academic profile demonstrated previous familiarity with educational technologies, making it easier to adapt to and interact with new digital tools in the teaching-learning context.

Although participants generally possessed adequate digital literacy from prior educational experiences, variability in digital proficiency levels could influence engagement and game effectiveness, which should be considered when generalizing results.

The participants had a mean age of 22.66 (SD 1.44; range 21-27) years. The participants unanimously agreed on the value of incorporating games into academic training and recognized the potential for learning through games (Table 1). The students used the application before they were in contact with their practice scenarios (supervised internship).

Students could download the app through a QR code, which optimized its access. The game takes approximately 35 minutes, so the researchers timed the students for 40 minutes to complete the task.

**Table 1.** Characteristics of the usability test participants (N=27).

Characteristics		Participants, n (%)	
Sex			
Female		20 (74.1)	
Male		7 (25.9)	
Semester			
Eighth		26 (96.3)	
Tenth		1 (3.7)	
Other experiences with ET^a^			
Yes		16 (59.3)	
No		11 (40.7)	
Do you like games?			
Yes, I like it a lot		18 (66.7)	
Yes, I like it a little		9 (33.3)	
I do not like it		0 (0)	
Do you think it is possible to learn through games?			
Yes		27 (100)	
No		0 (0)	
Would you like to include interactive games during your undergraduate course?			
Yes		27 (100)	
No		0 (0)	

a ET: educational technology.

### Validation

**Figure 3.**
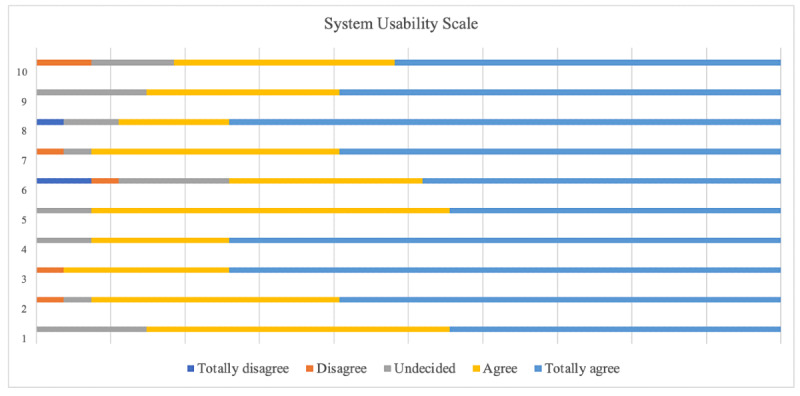
Evaluation of Health Unit in Focus application’s items using the System Usability Scale.

Based on the degree of agreement with the items evaluated using the SUS, which focused on assessing the suitability of the serious game as an educational tool on issues related to older adults’ health, the participants found the game easy to use, accessible, useful, and practical with well-founded content. The final score was 85.75, considered excellent, with a median of 86.57. All the other items scored >75, with a Cronbach α of 0.755 (Figure 3).

The items evaluated by SUS have specific usability characteristics with relevant meanings. Their scores have value intervals between 0 and 100 points to help quantify and qualify them, thus demonstrating software quality (Table 2). The score is classified as follows: worst imaginable (0‐20.5), poor (21‐38.5), average (39‐52.5), good (53‐73.5), excellent (74‐85.5), and best imaginable (86-100).

Based on individual participant responses, the serious game’s average usability value most frequently fell within the 86‐100 range on a scale from worst imaginable to best imaginable (Table 3).

**Table 2.** Usability of the “Health Unit in Focus” application as evaluated by the System Usability Scale (N=27).

Usability characteristics	Scores, median	Meaning
Ease of learning	89.34	Easy to understand
Efficiency	84.25	Agility in executing proposed tasks
Inconstancy	75.92	Absence or low number of errors
Ease of memorization	87.03	Easy-to-use system even after a period of inactivity
Satisfaction	86.11	Pleasant design
Total	84.53	Excellent

**Table 3.** Categorization and classification of the Health Unit in Focus (HUF) application usability using the System Usability Scale (SUS; N=27).

Categorization scale	Responses, n (%)	SUS classification
0‐20.5	0 (0)	Worst imaginable
21‐38.5	0 (0)	Poor
39‐52.5	1 (3.7)	Average
53‐73.5	2 (7.4)	Good
74‐85.5	9 (33.3)	Excellent
86‐100	15 (55.6)	Best imaginable

## Discussion

### Principal Findings

The effective use of educational technologies in the teaching-learning process is a hallmark of modern education, particularly in health education. Their impact lies in supporting personal, technical, and theoretical development through an engaging and enjoyable learning experience. In addition to this, these technologies optimize time, work, and even health care. During the training process, they can be used to prepare students for the field because clinical practice already uses several mobile technologies [23].

The development of the HUF serious game intends to facilitate access to everyday content on older adults’ health in a playful and interactive manner, and the target population validated it. The conditions added to this app describe possible clinical cases that appear daily in a nursing professional’s routine and are sometimes not covered in traditional classes. In this way, students may be more aware of how to approach these cases.

While results indicate strong usability and satisfaction within this specific academic setting, replication in diverse educational contexts, particularly those with varying degrees of technological infrastructure and student digital literacy, is essential for broader applicability.

It is essential that the development of new methodological resources, such as serious games, be based on scientific evidence to ensure greater reliability and success in their established objectives [24]. Studies show that educational games tend to achieve good results using standardized methods to meet educational demands [25,26]. Therefore, this study sought references previously used in the literature to construct and validate the HUF application [13,15].

Technological resources such as serious games must arouse students’ interest, be compatible with their needs, and suit (adapt to) their cognitive style. In this way, this technology can foster knowledge formation [27]. The HUF was developed based on feedback from nursing students, giving prominence to their most challenging subjects. We therefore sought to meet the needs of this audience.

Technologies should be engaging and enjoyable in addition to meeting user expectations. Combining leisure, engagement in the clinical context, and knowledge acquisition are key aspects of gamification and are recognized as essential elements of an enjoyable experience [28]. The proposed serious game incorporates these aspects, offering features such as totems, trophies, and opportunities for advancement and new achievements.

This approach demonstrates how technological resources facilitate the integration of theoretical aspects, practical daily life routines, and student accessibility [9].

Usability evaluation guarantees a system’s ease, suitability, and rapid learning in handling while seeking to minimize operational errors. Therefore, usability evaluation seeks greater efficiency, safety, and security in implementing such systems, making the user experience more pleasant [29].

According to Brooke [30], the aspects assessed by the SUS make it a reliable and robust evaluation tool with a high degree of validity. They are essential for a good software result, covering a range of system aspects such as complexity, need for support, interface, and others.

Regarding these aspects, the HUF serious game demonstrated good usability, as the overall score was considered excellent. HUF’s highest score was in the odd-numbered items that deal with positive aspects found in the analysis. Other findings present a variation in usability between 78.8 and 88.2 among young students [31-33].

There is a constant growth in the use of serious games as a learning tool in the health sector. These digital games, developed both for educational and entertainment purposes, have been widely used to improve the theoretical and practical knowledge of students and health professionals. Several studies have shown that serious games can improve knowledge retention, clinical skills development, and decision-making, making them a promising pedagogical resource for training more prepared and qualified professionals [34,35].

The SUS is a widely adopted tool for measuring the usability of various software and hardware products. However, its original conception was not specifically aimed at evaluating mobile apps. However, studies have shown its effective use with promising results [36,37].

A study carried out in Brazil evaluated the usability of a digital tool using SUS. The study reported a usability score of 75 [38]. Another study, carried out in Germany, validated a virtual reality serious game for patients. The SUS results showed a final score of 86.9 [39]. These studies are in line with the findings reported in this paper.

The scale used also allows for assessing user satisfaction, which is considered a key component of usability [30]. The HUF demonstrated high user satisfaction, with a score 86 on items 1, 4, and 9, supporting this assessment. Previous studies have shown that the use of serious games is associated with similarly high satisfaction rates [40-42].

The modern scenario involves technologies in the daily lives of the entire population, especially young people and adults. This involvement has brought new needs and challenges to producing relevant and acceptable educational products. In this sense, the researcher must be able to transform knowledge into attractive, coherent, and intriguing products for the user [43]. According to the participants’ evaluation, HUF proved to be a pleasant and easy-to-use system with low errors.

Integrating innovative tools and strategies into education and training is not a novel concept. However, combining new dynamic and interactive approaches is critical for enhancing learning [44]. In addition to the educational or training process, technological advancements have resulted in higher-quality nursing care in a variety of contexts [45-47].

Games, especially serious games, are advantageous for undergraduate health courses, positively impacting student motivation and satisfaction. As educators, teachers should be attentive to complementing learning methods with resources such as games as long as they are reliable and validated [48]. Notably, the HUF serious game has reliable internal consistency and has been appropriately validated.

### Limitations

This study has several limitations. The application use time was approximately 40 minutes, which could limit its depth if not integrated into the participants’ regular academic routine. Immediate access also depended on a stable internet connection or a prior download. The small sample size and the fact that it was conducted with students in a single center are notable limitations. The study did not include before or after testing or comparisons with other instructional formats. As no other studies have addressed this topic to date, the potential influence of the novelty effect on the results remains unexplored. Lastly, functional technical limitations, such as the lack of communication between synchronous players, may have influenced the outcomes.

Future studies should incorporate longitudinal designs, multicenter participation, and comparative evaluations against traditional and alternative digital education formats to better evaluate long-term effectiveness and transferability of learned skills.

### Conclusions

In the current scenario, characterized by rapid technological advancements and easy access to information, the challenge of enhancing the educational process, particularly in the health sector, is significant. Developing technological tools that align with the target audience’s needs can enhance the learning process. The HUF serious game was validated with undergraduate students, with promising results for its use and potential replication in similar contexts. Furthermore, HUF features easy access to information, a user-approved interface, and content relevant to older adults’ health, making it a valuable educational resource.

## Supplementary material

10.2196/66289 Multimedia Appendix 1 Examples of clinical cases.

10.2196/66289 Multimedia Appendix 2 Responses to the educational games evaluation model.

## Abbreviations

GAMING: Game-Based Intervention Reporting Guidelines
HUF: Health Unit in Focus
PHC: primary health care
SUS: System Usability Scale
