# Supplementary material for: A Serious Game (Health Unit in Focus) for Enhancing Undergraduate Education on Older Adults’ Health: Design and Validation Study
Source: JMIR Serious Games. 2025 Nov 4;13:e66289. doi: 10.2196/66289 (PMC12584992; doi:10.2196/66289)
Supplement: Multimedia Appendix 1 [file games-v13-e66289-s001.docx]

Multimedia Appendix 1

| **Questions** | |
| --- | --- |
|  |  |
| 1 | Mr. Carlos, 64 years old, has long-term metabolic syndrome and insulin resistance. He makes daily and continuous use of insulin, which can cause adverse effects such as insulin lipodystrophy. This condition is characterized by fibrous and little vascularized lesions in subcutaneous adipose tissue. This case is RIGHT or WRONG. |
| 2 | Estela has diabetes and uses insulin continuously and regularly at home. When necessary, the concomitant administration of the two types of insulin was oriented so that it would first be aspirated to NPH insulin and then regular. This case is RIGHT or WRONG. |
| 3 | David, 58, has diabetes and continuously uses insulin. As usual, he went to the Basic Health Unit (UBS) to seek his continuous medications. Upon checking the medical record, the team verified the prescription of 12 regular human insulin units (applied between 30 and 45 minutes before meals start) subcutaneously twice daily. The unit has insulin 100 IU/ml in 5 ml vials. David will receive two vials to use at home for a thirty-day treatment. This case is RIGHT or WRONG. |
| 4 | Betina, 67, recently lost her mother due to complications from type 2 diabetes. Following this incident, she resolved to take care of herself, requesting routine tests and consulting with health professionals. One of her worries was the occurrence of diabetes and other chronic nontransmittable diseases due to inheritance. During the consultation, it was stated that the factors contributing to these syndromes' pathogenesis are exclusively acquired; that is, they are only related to the person's lifestyle. This case is RIGHT or WRONG. |
| 5 | Mariana, a 58-year-old obese woman, was diagnosed with metabolic syndrome (7 years ago) and diabetes (2 years ago). The accumulation of these clinical health conditions impacts her social, physiological, and endocrine context. Nevertheless, her inflammatory parameter, as measured by PCR examination (0.3 - 1), remains unchanged. Thus, activation of the inflammatory process is unrelated to the clinical conditions of metabolic syndrome, obesity, and diabetes. This case is RIGHT or WRONG. |
| 6 | Gabriela, 68, is a widow who has been hypertensive for more than a decade. She underwent an elective surgical procedure and, during preoperative hospitalization, found that she possessed the correct number of components required for metabolic syndrome diagnosis. The Surgical Center’s multi-professional team clarified the existing risks of metabolic syndrome and systemic arterial hypertension, which could happen during the procedure. Therefore, the risk of mortality due to hypertension is independent, even though it is one of the components of metabolic syndrome. This case is RIGHT or WRONG. |
| 7 | Valter, 68, has had high blood pressure for a long time and is being monitored at the Basic Health Unit near his home. Upon arriving at the unit, he was told that his vital signs would be checked. When measuring blood pressure, the patient’s positioning and behavior are essential to avoid interfering with the result. The health professional must verify the following information: the patient’s bladder is not full; the patient has not consumed coffee, alcoholic beverages, or food before the measurement; the patient has not exercised in the last 60 minutes and has not smoked in the last 30 minutes. Regarding the information checked, this case is RIGHT or WRONG. |
| 8 | Flávia, a 72-year-old black woman with grade II obesity and a history of systemic arterial hypertension, was admitted to the hospital emergency department with epigastric pain and dyspnea on mild exertion. As part of the initial care, an electrocardiogram was requested. To perform this exam, the healthcare professional must have prior knowledge of the cardiac foci, which are aortic, pulmonary, tricuspid, valvular, and mitral foci. Regarding the cardiac foci, this case is RIGHT or WRONG. |
| 9 | Insulin resistance is one component of Metabolic Syndrome. It is known that isolated factors do not accurately diagnose the syndrome; rather, a combination of these factors is necessary. IInsulin resistance predisposes individuals to the onset of hypertension, which develops from the impairment of the venous network and its vasodilatory and vasoconstrictive action. This case is RIGHT or WRONG. |
| 10 | Rafaela, a 69-year-old woman with a family history of chronic diseases, lost her parents to complications from Systemic Arterial Hypertension. Aware of her risk factors, she decided to change her routine and lifestyle habits. Her transformation included monitoring by a health professional who could guide her eating habits. Measures such as low intake of saturated fats, trans fats, cholesterol, sodium, and sugars are part of essential basic guidelines to help exclusively with hypertension. This case is RIGHT or WRONG. |
