# Supplementary material for: A Serious Game (Health Unit in Focus) for Enhancing Undergraduate Education on Older Adults’ Health: Design and Validation Study
Source: JMIR Serious Games. 2025 Nov 4;13:e66289. doi: 10.2196/66289 (PMC12584992; doi:10.2196/66289)
Supplement: Multimedia Appendix 2 [file games-v13-e66289-s002.docx]

Multimedia Appendix 2

| Responses to the educational games evaluation model. Brasília, 2025. | | | | | |
| --- | --- | --- | --- | --- | --- |
|  | **Mean** | **SD** | **P25** | **Median** | **P75** |
| **Motivation** |  |  |  |  |  |
| 1. The game design is appealing | 4.4 | 0.2 | 4.0 | 5.0 | 5.0 |
| 2. Something was interesting at the beginning of the game that caught my attention | 4.5 | 0.1 | 4.0 | 5.0 | 5.0 |
| 3. The variation (form, content, or activities) helped me stay focused on the game | 4.5 | 0.2 | 4.0 | 5.0 | 5.0 |
| 4. The game content is relevant to my interests | 4.8 | 0.1 | 5.0 | 5.0 | 5.0 |
| 5. The way this game works is suited to my learning style | 4.2 | 0.3 | 4.0 | 4.0 | 5.0 |
| 6. The game content is connected to other knowledge I already had | 4.5 | 0.1 | 4.0 | 5.0 | 5.0 |
| 7. It was easy to understand the game and start using it as study material | 4.5 | 0.2 | 4.0 | 5.0 | 5.0 |
| 8. As I went through the stages of the game, I felt confident that I was learning | 4.5 | 0.1 | 4.0 | 4.0 | 5.0 |
| 9. I am satisfied because I know that I will have opportunities to put into practice things I learned from the game | 4.5 | 0.2 | 4.0 | 5.0 | 5.0 |
| 10. It is because of my personal effort that I am able to advance in the game | 4.0 | 0.2 | 4.0 | 4.0 | 4.0 |
| **User Experience** |  |  |  |  |  |
| 11. I temporarily forgot my daily worries; I was totally focused on the game | 3.8 | 0.3 | 3.0 | 4.0 | 5.0 |
| 12. I didn’t notice time passing while playing when I saw the game was over | 3.5 | 0.3 | 2.0 | 4.0 | 4.0 |
| 13. I felt more in the game environment than in the real world, forgetting what was around me | 3.2 | 0.3 | 2.0 | 3.0 | 4.0 |
| 14. I was able to interact with other people during the game | 3.3 | 0.3 | 2.0 | 4.0 | 4.0 |
| 15. I had fun together with other people | 3.1 | 0.2 | 2.0 | 3.0 | 4.0 |
| 16. The game promotes moments of cooperation and/or competition between the people who participate | 3.4 | 0.4 | 2.0 | 3.0 | 5.0 |
| 17. This game is adequately challenging for me; the tasks are neither too easy nor too difficult | 3.9 | 0.3 | 4.0 | 4.0 | 5.0 |
| 18. The game evolves at an adequate pace and does not become monotonous - it offers new obstacles, situations, or variations of activities | 3.6 | 0.4 | 2.0 | 4.0 | 5.0 |
| 19. I had fun with the game | 4.3 | 0.2 | 4.0 | 4.0 | 5.0 |
| 20. When interrupted, I was disappointed that the game was over | 2.8 | 0.3 | 2.0 | 3.0 | 3.0 |
| 21. I would recommend this game to my colleagues | 4.8 | 0.1 | 5.0 | 5.0 | 5.0 |
| 22. I would like to use this game again | 4.6 | 0.2 | 4.0 | 5.0 | 5.0 |
| 23. I was able to achieve the objectives of the game through my skills | 4.3 | 0.1 | 4.0 | 4.0 | 5.0 |
| 24. I had positive feelings of efficiency in the course of the game | 4.4 | 0.1 | 4.0 | 4.0 | 5.0 |
| 25. The controls for performing actions in the game responded well | 4.0 | 0.3 | 4.0 | 4.0 | 5.0 |
| 26. It is easy to learn how to use the game’s interface and controls | 4.8 | 0.1 | 5.0 | 5.0 | 5.0 |
| **Learning** |  |  |  |  |  |
| 27. The game contributed to my learning about NCDs and older adult health | 4.8 | 0.1 | 5.0 | 5.0 | 5.0 |
| 28. The game was efficient for my learning compared to other activities I have had on NCDs and older adult health | 4.6 | 0.2 | 4.0 | 5.0 | 5.0 |
| 29. The experience with the game will contribute to my performance in my professional life | 4.5 | 0.2 | 4.0 | 5.0 | 5.0 |

**Abreviaturas:** DP, Desvio Padrão; P25, Percentil 25; P75, Percentil 75.

**Abbreviations:** SD, Standard Deviation; P25, 25th Percentile; P75, 75th Percentile; NCDs, Noncommunicable chronic diseases

Fonte: Dados da Pesquisa

**Source:** Survey Data
